# A Patient With Dengue Fever and COVID-19: Coinfection or Not?

**DOI:** 10.7759/cureus.11955

**Published:** 2020-12-07

**Authors:** Afnan A Malibari, Faisal Al-Husayni, Abdullah Jabri, Abdulfattah Al-Amri, Maher Alharbi

**Affiliations:** 1 Internal Medicine, King Saud Bin Abdulaziz University for Health Sciences, Jeddah, SAU; 2 Internal Medicine, National Guard Hospital, King Abdulaziz Medical City, Jeddah, SAU; 3 Internal Medicine, King Abdullah International Medical Research Center, Jeddah, SAU; 4 Department of Pathology and Laboratory Medicine, King Abdullah International Medical Research Center, Jeddah, SAU; 5 Infection Prevention and Control, King Abdulaziz Medical City, Jeddah, SAU; 6 College of Medicine, King Saud Bin Abdulaziz University for Health Sciences, Jeddah, SAU; 7 Infectious Diseases, King Abdullah International Medical Research Center, Jeddah, SAU

**Keywords:** co-infection, covid-19, dengue fever, dengue virus infection, epidemic, healthcare system, pandemic, public health, sars-cov2

## Abstract

Co-epidemics can create a burden on healthcare systems in the affected areas. The world, at present, is facing the pandemic of coronavirus disease. Nonetheless, many areas worldwide suffer from endemics that are not of less danger than the current pandemic. We presented a case of a patient diagnosed with dengue fever and was also found to have coronavirus through nasal swab, but immunoglobulin M and G were undetectable. Our case brings to notice the alarming probability of two co-epidemics happening simultaneously. However, through the presented case, our theory is that the dengue virus may cause a false-positive detection of severe acute respiratory syndrome coronavirus 2.

## Introduction

Novel coronavirus disease 2019 (COVID-19) is a respiratory illness caused by severe acute respiratory syndrome coronavirus 2 (SARS-CoV-2). It was first reported as an outbreak in Wuhan, China, and spread worldwide, causing a pandemic disease [1]. Symptoms of COVID-19 can range from mild symptoms of fever, cough, headache, muscular pain, nausea, and vomiting to a severe illness causing pneumonia, acute respiratory distress syndrome (ARDS), septic shock, and multi-organ failure [2]. Simultaneously, the dengue virus (DENV) transmitted by Aedes aegypti and Aedes albopictus is an endemic disease in the western area of Saudi Arabia [3]. DENV presents with fever, muscular pain, malaise, and rash, which makes it difficult to distinguish between dengue fever and SARS-CoV-2 infection [4]. A co-infection of SARS-CoV-2 and DENV has not been well studied. It is unclear if it can coexist with DENV in the same affected person, as it was assumed that DENV could block the entry of another virus in the same host cell [5]. In another study, a blockage of angiotensin II type 1 receptor (AT1 receptor) by losartan, and angiotensin I-converting enzyme (ACE) by enalapril in mice infected with DENV, showed a reduction of DENV entry [6]. It is still unclear if SARS-CoV-2 can block the entry of DENV by inhibiting the ACE receptors. In this report, we present a case of a patient with a positive dengue serology, and detectable dengue nonstructural protein-1 (NS1) antigen and COVID-19 diagnosed by reverse transcription-polymerase chain reaction (RT-PCR). 

## Case presentation

**Figure 1:**
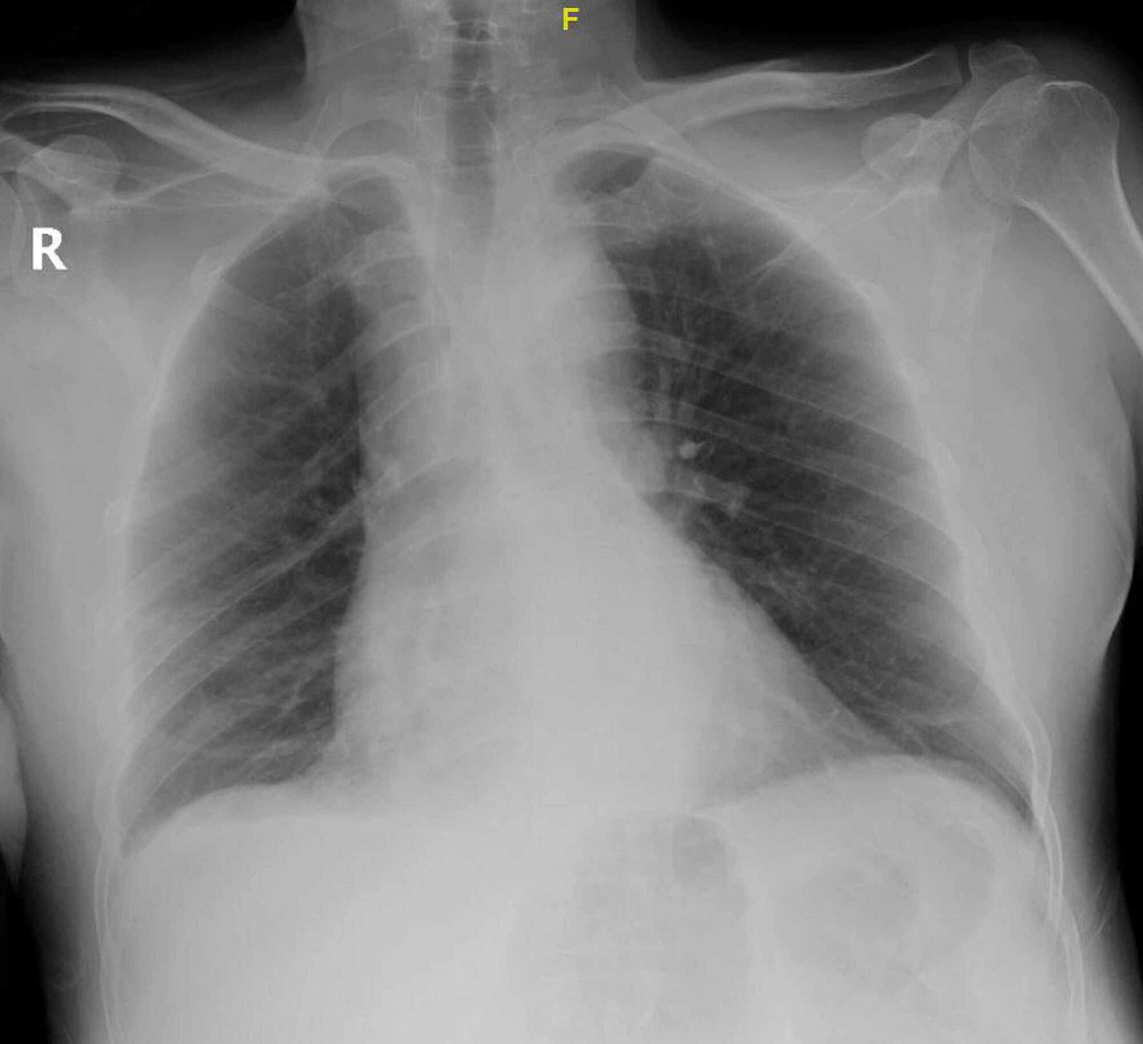
Patient’s chest image showing bilateral atelectasis and small right effusion

A 58-year-old male with no significant past medical history presented to the hospital with fever, malaise, and generalized body aches. The patient was exposed to multiple mosquito bites ten days before presentation. Four days later, the patient started to experience symptoms. He had no respiratory or gastrointestinal symptoms. He also denied a history of contact with COVID-19 patients. Upon presentation, his vital signs showed a low-grade fever of 37.7 C, blood pressure of 116/69 mm Hg, heart rate of 64 beats per minute, respiratory rate of 18 breaths per minute, and maintaining oxygen saturation 99% while breathing ambient air. Lung and cardiac auscultation were normal apart from bilateral fine basal crepitations. A complete blood count revealed severe thrombocytopenia of 17^x109/L^, white blood count (WBC) of 4.5^x109/L^, lymphocytes count of 2.5^x109/L^, and neutrophils count of 1.63^x109/L^, while his renal and liver profiles were within normal ranges. His chest images showed bilateral atelectasis and small right effusion (Figure 1).

On the next day, dengue serology came back positive, including immunoglobulin G (IgG), immunoglobulin M (IgM), nonstructural protein 1 (NS1) antigen. Additionally, as a part of the hospital’s protocol to screen all patients for COVID-19, the SARS-CoV-2 virus was detected through the nasal swab. Four days later, COVID-19 antibody testing was sent and came back negative with an IgM and IgG of less than 1 AU/mL. During his hospitalization, the patient had minor gum bleeding, requiring a platelet transfusion. After that, he was stable throughout his stay without symptoms recurrence, and platelets count recovered. The patient was discharged after completing seven days in the hospital in good condition.

## Discussion

In dengue-endemic countries, healthcare providers faced challenges to initially distinguish COVID-19 from dengue as they both exhibit non-specific presentations, including fever, headache, abdominal pain, malaise, and nausea. Not only clinical features, but they also share laboratory findings such as leukopenia and thrombocytopenia, which put more stress on healthcare workers to combat [7]. Our case report describes a patient admitted to the hospital as a dengue fever patient meeting the diagnostic criteria of the disease clinically and laboratory. Due to the pandemic of COVID-19, the protocol in most hospitals includes the screening of patients for SARS-CoV-2 through nasopharyngeal swab. In addition to positive dengue, our patient showed a positive COVID-19 PCR result ( RealStar® SARS-CoV-2 RT-PCR Kit 1.0, Altona Diagnostics, Hamburg, Germany) in the screening swab but had negative IgG and IgM using Liaison Sars-CoV-2 S1/S2 (DiaSorin, Saluggia, Italy) for COVID-19 in the sample done on day 12 post-symptoms.

A variety of explanations have been proposed to clarify such a scenario. Studies demonstrated a possible role of angiotensin and angiotensin II converting enzyme (ACE2) in the pathogenesis of different viruses like H7N9 influenza, SARS coronavirus, and dengue virus. An animal study proved that the in-vivo blocking of angiotensin II type 1 receptor (AT1 receptor) by losartan and the inhibition of ACE using enalapril led to a reduction in the percentage of macrophages expressing DENV [6]. Their conclusion, combined with the fact that ACE2 can also facilitate SARS-CoV2 entry, suggested a viral interference of SARS-CoV-2 over the dengue virus [8]. RT-PCR is one of the major tests used for diagnosing viral infections. This molecular method is highly specific as it works by detecting the distinctive genetic sequence of the viral RNA presented in the collected sample. Centers for Disease Control and Prevention (CDC) recommended the nasopharyngeal swab as a good sample, which contains enough amount of the viral material required for the COVID-19 molecular recognition [9]. Despite the high accuracy of PCR, many factors can affect the test sensitivity, including the timing and technique of obtaining the sample. The early onset sample and the inappropriate way of the collection were the main contributors to the highly documented false-negative results for SARS-CoV-2 in RT-PCR [5, 9].On the other hand, false-positive results in genetic testing are rare but can happen. The false-positive results are mainly due to human errors such as contamination of the swab from the lab where it was handled and processed or from the PCR machine that was contaminated with a previous positive sample [10]. Also, false-positive SARS-CoV-2 results could occur when there is a cross-reaction with other related viruses such as rhinoviruses or other coronaviruses strains [9, 10]. The sensitivity of the PCR to detect even small amounts of viral RNA or dead viral fragments from the previous infection in the convalesced COVID-19 patients, which might consequently produce a false-positive result [11].

Another way to diagnose viral infections is to detect the antibodies produced in the patient's blood against the invading virus. In dengue fever, commercial serological kits are available for identifying the IgM anti-DENV [12]. Nonetheless, a high rate of a false-positive result of enzyme-linked immunosorbent assay (ELISA; PanBio IgM or IgG) was reported in a European study when only one serum sample was used for diagnosing dengue, and a second and more specific test was required to confirm it [13]. In the absence of the second test, a combination of thrombocytopenia and leukopenia findings was suggestive for the diagnosis, as it has been found in 40.4% of the confirmed dengue cases.

Another disadvantage of the dengue serological testing was found in countries suffering from both dengue fever and COVID-19. Studies conducted in those countries documented a false-positive dengue IgM in confirmed COVID-19 patients highlighting the possibility of antigenic similarity between those viruses in which SARS-CoV-2 may trigger the production of anti-DENV antibodies by the immunological memory cells [14]. Furthermore, other studies hypothesized that SARS-CoV-2 antibodies might cross-react with DENV antigens used in the dengue serological tests causing misleading results [15, 16]. Regarding COVID-19, currently available data illustrated that infected patients might display a detectable IgM between day 10 and day 21 post-infection, and the average seroconversion time for IgM and IgG appears at day 11, day 12, and day 14, respectively [17, 18]. Thus, the early phase of the disease may give false-negative antibody results. Antibody's production in mild cases may take longer than this average time, and in some cases, they were not detected at all [19].

The variety of developing antibodies and the delayed response of the body's immunity toward COVID-19 are still not fully understood. From all the above information, our case report raises questions regarding the sensitivity and specificity of the molecular and serological diagnostic tests used for DENV and COVID-19 infections. It also emphasizes the need for further deep cellular and immunological studies to understand whether the presence of both viruses can affect antibodies' response. Understanding such mechanisms will help avoid incorrectly interpreting the positive and negative results and prevent the fatal outcomes resulting from the misdiagnosis of both viruses [4]. However, a number of viruses have been found to cause infections simultaneously, even with SARS-CoV-2 [5, 20]. Thus, due to the low sensitivity of the serological tests, our case-report could indicate a potential coinfection of dengue fever and COVID-19.

## Conclusions

In this case, we presented a patient with dengue fever and COVID-19 that triggers an alert in dengue-endemic countries. Facing these viruses, which share many similarities in the symptoms, warrant governments to raise awareness about avertable causes such as mosquito control programs. Also, as our patient had a positive swab and undetectable IgG and IgM, there is a possibility of DENV to cause false-positive results of SARS-CoV-2, which suggests further studies to confirm or overrule this theory.
