# Correction: A Patient With Dengue Fever and COVID-19: Coinfection or Not?

**DOI:** 10.7759/cureus.c381

**Published:** 2025-11-18

**Authors:** Afnan A Malibari, Faisal Al-Husayni, Abdullah Jabri, Abdulfattah Al-Amri, Maher Alharbi

**Affiliations:** 1 Internal Medicine, King Saud Bin Abdulaziz University for Health Sciences, Jeddah, SAU; 2 Internal Medicine, National Guard Hospital, King Abdulaziz Medical City, Jeddah, SAU; 3 Internal Medicine, King Abdullah International Medical Research Center, Jeddah, SAU; 4 Department of Pathology and Laboratory Medicine, King Abdullah International Medical Research Center, Jeddah, SAU; 5 Infection Prevention and Control, National Guard Hospital, King Abdulaziz Medical City, Jeddah, SAU; 6 Infection Prevention and Control, King Abdullah International Medical Research Center, Jeddah, SAU; 7 Saudi Arabia Infectious Diseases, King Abdullah International Medical Research Center, Jeddah, SAU

The affiliations for Maher Alharbi have been corrected to:

1. Infection Prevention and Control, King Abdulaziz Medical City, Jeddah, Saudi Arabia
2. College of Medicine, King Saud Bin Abdulaziz University for Health Sciences, Jeddah, Saudi Arabia
3. Saudi Arabia Infectious Diseases, King Abdullah International Medical Research Center, Jeddah, Saudi Arabia

